# The Evolution of a Capacity to Build Supra-Cellular Ropes Enabled Filamentous Cyanobacteria to Colonize Highly Erodible Substrates

**DOI:** 10.1371/journal.pone.0007801

**Published:** 2009-11-17

**Authors:** Ferran Garcia-Pichel, Martin F. Wojciechowski

**Affiliations:** School of Life Sciences, Arizona State University, Tempe, Arizona, United States of America; Universidad Miguel Hernandez, Spain

## Abstract

**Background:**

Several motile, filamentous cyanobacteria display the ability to self-assemble into tightly woven or twisted groups of filaments that form macroscopic yarns or ropes, and that are often centimeters long and 50–200 µm in diameter. Traditionally, this trait has been the basis for taxonomic definition of several genera, notably *Microcoleus* and *Hydrocoleum*, but the trait has not been associated with any plausible function.

**Method and Findings:**

Through the use of phylogenetic reconstruction, we demonstrate that pedigreed, rope-building cyanobacteria from various habitats do not form a monophyletic group. This is consistent with the hypothesis that rope-building ability was fixed independently in several discrete clades, likely through processes of convergent evolution or lateral transfer. Because rope-building cyanobacteria share the ability to colonize geologically unstable sedimentary substrates, such as subtidal and intertidal marine sediments and non-vegetated soils, it is also likely that this supracellular differentiation capacity imparts a particular fitness advantage in such habitats. The physics of sediment and soil erosion in fact predict that threads in the 50–200 µm size range will attain optimal characteristics to stabilize such substrates on contact.

**Conclusions:**

Rope building is a supracellular morphological adaptation in filamentous cyanobacteria that allows them to colonize physically unstable sedimentary environments, and to act as successful pioneers in the biostabilization process.

## Introduction

**Figure 1:**
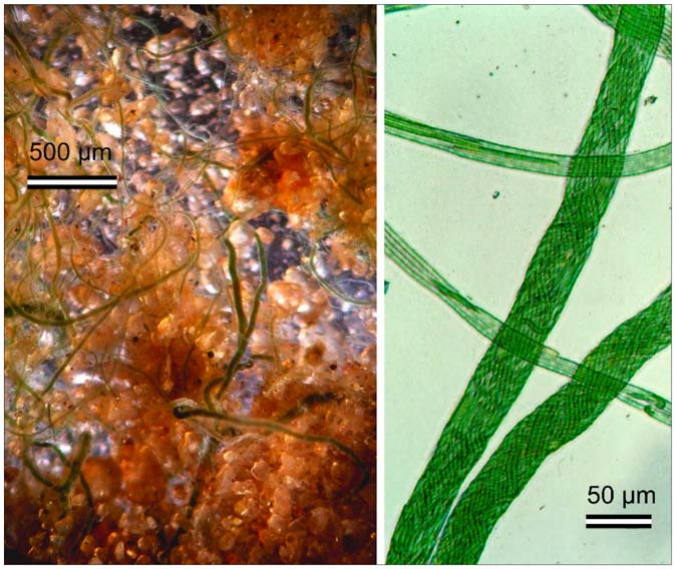
Rope-building in cyanobacteria. Photomicrograph of a sandy desert soil from the Colorado Plateau colonized by pioneering *Microcoleus vaginatus* with visible large ropes spanning the quartz sand grains (left). A microscopic view of ropes built in culture by an isolate of *Microcoleus chthonoplastes* from a marine intertidal mat. The single filaments are clearly visible in the photograph.

It has long been known that certain filamentous cyanobacteria can form tightly-woven, rope-like bundles of trichomes (cohesive rows of cells) that remain held together in a common, tubular, extracellular polysaccharide sheath [1], [2], even though this trait has not been related to any particular ecophysiological function (Fig. 1). It has, however, been used in taxonomy as the main morphological character to define several traditional genera, such as *Microcoleus*, *Schizothrix* or *Hydrocoleum* [3], with the tacit phylogenetic implication that rope-building is a synapomorphy, a case of shared inheritance of this trait in the present-day descendants of a common ancestor that built such ropes. While the genetic basis of rope-building has not been studied directly, due to the lack of genetically modifiable strains, it is likely a genetically controlled trait, inasmuch as it appears easily lost in spontaneous genetic variants upon continued cultivation [4]. We use the term *rope* to denote the presence of tighly woven filaments in the multifilament struture, as a special case of the more inclusive terms *bundle* or *fascicle*, that only imply a multiplicity of filaments more or less tightly held together by a common sheath, and would include cyanobacterial general like *Trichodesmium* or *Aphanizomenon*.

Rope-building cyanobacteria today thrive in habitats as disparate as desert soils [5], [6], marine subtidal stromatolites [7], [8], and intertidal sediments [4]. In natural habitats, multifilament ropes are the preferred configuration, and single filaments or trichomes are rare. The nature of the evolutionary advantage conferred by rope-building, however, is all but evident. In fact, according to size-scaling models, growth as large aggregated bundles instead of separate, single trichomes, must certainly bring about at least some negative physiological effects. For example, self-shading effects among the filaments in a bundle configuration will decrease significantly the overall photosynthetic efficiency with respect to incident radiation, and these effects become particularly noticeable as cyanobacteria reach sizes larger than some 10 µm in diameter [9]. Nutrient and metabolite exchange is generally thought to be the main constraint for attaining large sizes in organisms without internal transport systems [10]. A bundle configuration is likely to hamper the efficiency of nutrient uptake systems, and will likely create significant local accumulation of metabolic by products, such as the photosynthetically-derived molecular oxygen. It is thus only logical to expect some trade-off, an advantage conferred by rope-building determinant enough as to overcome the competitive burdens brought about by crowding.

We contend that the correct interpretation of the rope-building phenomenon can be informed by knowledge of the evolutionary history of the organisms that display this faculty. Particularly if the phylogenetic distribution of the trait indicates that it was gained, and retained, independently in several evolutionarily distant groups of cyanobacteria, then one can seek evidence for the nature of its adaptive value in environmental characteristics that are common to the habitats typical of those particular groups, but absent in those lacking it. Once this is achieved, and as a means to validate the correlation, one should seek functional models that explain the fitness value of the phenotypic trait in terms of a mechanism exclusively relevant to the shared environments. We used a phylogenetic investigation of pedigreed strains or field samples of demonstrated rope-builders to explore this hypothesis, and provide a fitness value model based on the application of sedimentary physics to this phenomenon.

For the initial phylogenetic investigation, at least three alternative hypotheses can be considered, the implications of which are quite different. If all rope-building cyanobacteria form a coherent monophyletic group, this implies that the trait evolved only once and its continued presence in a variety of habitats may be related to its fitness value, but it may also be a simple legacy of shared ancestry, similar to the fact that the exact number of legs in tetrapods is four. Any inferences made from this situation would be weak (as one cannot infer that four legs are required for locomotion). Alternatively, we may find that rope-builders constitute a polyphyletic group, distributed in two or more, well-separated clades. In this case two explanations are possible. In one, rope-building appeared in the common ancestor of all filamentous cyanobacteria, but was subsequently lost in one or more lineages over time as they evolved and adapted to different habitats. This situation would not allow us to distinguish strictly between fitness value vs. evolutionary legacy either, precluding further inferences. A second, simpler explanation for polyphyly, is that the trait was not inherited from a common ancestor, but rather gained independently in each clade during the course of cyanobacterial evolution. In this scenario, evolutionary legacy can play no role in determining the trait's distribution, only positive selection. Here one can seek evidence for the possible fitness value of rope building in environmental characteristics common to the rope-building clades, but absent in other cyanobacteria. We note that two distinct evolutionary mechanisms may result in the independent gain of a trait: horizontal gene transfer or convergent evolution. Simple phylogenetic analyses cannot easily distinguish between them. The inference about the need for positive selection is the same, however, regardless of the mechanism.

## Results and Discussion

### Polyphyly of rope building

**Figure 2:**
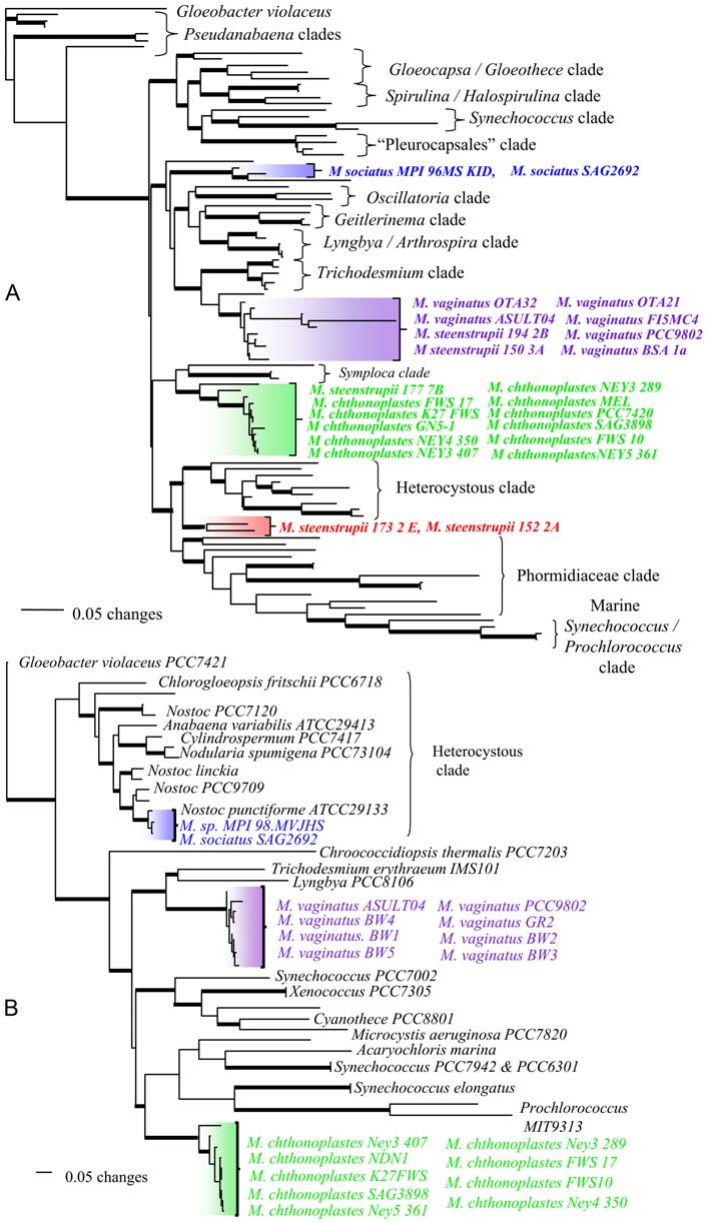
Phylogenetic relationships of *bona-fide*, rope-building cyanobacteria based on 16S rRNA (A) and *kai* C (B) sequences. Topologies shown are a Bayesian consensus of 200 trees sampled from stationarity (post burnin) derived from each analysis. Branches with posterior probabilities >0.95 are indicated by bold lines; all other branches have posterior probabilities of 0.50–0.95. Different, congruent clades of rope-builder are indicated in color. *Gloeobacter violaceus* was designated as the outgroup for all analyses. Entries are not all labeled for clarity, and some clades are given names based on the overall composition. Complete trees are available as additional information. Results from maximum parsimony analyses of both the 16S rRNA and *kaiC* sequences were largely congruent with Bayesian analyses shown here, although many clades receive only low to moderate support by non-parametric bootstrap analysis in the most parsimonious trees identified (data not shown).

To test our hypothesis that rope-building evolved independently in several groups of cyanobacteria, we reconstructed the phylogenetic relationships of *bona fide* rope-building strains and field samples based on sequence comparisons of two genes (16S rRNA and *kaiC*), newly obtained for this work or from traceable sequences available in public databases. These two genes were chosen because the number of sequences available from other genes in relevant cyanobacterial representatives is very limited. We focused our analyses on rope-building cultures and field samples from intertidal marine microbial mats and desert soils, for which we had the largest database coverage, and for which we had easy access to field samples. Our choice was based on the logical tenet that if polyphyly could be demonstrated in any one subgroup of rope-builders, then the results would be necessarily applicable to the whole. Bayesian analyses of 16S sequences obtained from cultures and field samples from terrestrial and marine origin, show that rope formers are indeed polyphyletic, and resolved into several separate, distinct, and statistically well-supported clades within the cyanobacterial phylogenetic tree (Fig. 2a). While these correspond roughly, if not absolutely, to traditional taxonomic entities, one should resist the temptation of nomenclatural distractions: the phylogenetic analysis clearly supports the fact that pedigreed rope-builders are polyphyletic. In fact, to propose that the two main clades (purple and green in Fig. 2a) are monophyletic would require one to break six nodes with statistical support >0.9 each (Bayesian posterior probabilities), a scenario with a combined probability <1/10^6^. Having established this point, its taxonomic implications may be noteworthy. Our results are in line with findings of previous studies [4] and a recent report that proposes a revision of the genus *Microcoleus* into at least two genera based on analyses of molecular, morphological, and structural characters [11]. We can clearly extend the need for revision to the species *M. steenstupii* and possibly *M. sociatus*.

The most logical explanation for this 16S rRNA tree is that that rope building among cyanobacteria has evolved independently at least in each of these four clades. However, the ancient trait explanation, where the common ancestor of all of these distinct, well-defined clades possessed the trait but it was repeatedly lost through evolution in most intervening lineages, cannot be rejected as such. Because of the large evolutionary distance spanned by the clades of rope-builders, this would require that rope building was a truly ancestral trait dating back to the early diversification of the filamentous cyanobacteria.

In order to find independent confirmation of polyphyly and to narrow our interpretations, we used a second, independent marker, *kaiC*, from a more limited set of samples. The *kaiC* gene is a member of a small cluster of genes that are important for maintaining circadian rhythms in cyanobacteria [12], [13]. In general, distinct, well-resolved, and supported clades were also found for strains and field samples of the terrestrial *M. vaginatus* and the marine *M. chthonoplastes* based on Bayesian analyses of this gene (Fig. 2b), a result congruent with our initial explanation of polyphyly based on 16S sequences. In the case of *M. sociatus*, we note the possibility of a case of horizontal transfer of *the marker gene*, since the two sequences of *kaiC* available from this species are clearly nested within a clade otherwise comprised of the more distantly related heterocystous cyanobacterium *Nostoc* (Fig. 2b). To propose a monophyletic origin of the two major clades would require breaking 5 well-supported nodes (probability <1/10^6^). The *kai*C tree however, offered an interesting additional piece of information: the ancient trait interpretation would require postulating that a very ancient, rope-building, filamentous, ancestor existed that gave rise to most extant clades of cyanobacteria. It also would necessitate that a large and diverse group composed exclusively of unicellular or colonial, but not filamentous, cyanobacteria (*Synechoccoccus*, *Xenococus*, *Acaryochloris*, *Microcystis*; see Fig. 2b) had a filamentous ancestor that made ropes, which is logically untenable.

Thus, our combined analyses speak for a scenario where either through convergent evolution or horizontal transfer (or both) across already well-separated evolutionary lineages, rope-building ability has been retained in phylogenetically separate groups of filamentous cyanobacteria, presumably by offering fitness contributions important enough. The main implication of this finding for our work is that the fitness value of rope building has to do with some ecological constraint(s) shared by these ecologically diverse cyanobacteria.

### A common denominator

A survey of the literature reveals what is common to all of these rope-builders: they are pioneers in the colonization of unstable sedimentary substrates such as sandy soils (*M. vaginatus* [5], [14], *M. steenstrupii* [15], *M. sociatus* [6]), intertidal sand flats (*M. chthonoplastes* [5], [13]), and subtidal marine carbonate sediments (*Schizothrix* spp. [8], *Hydrocoleum cantharanidosum* [16]). It is important to point out that these are not the only microorganisms that can stabilize unconsolidated sediments, since many different microbes are well-known biostabilizing agents in sedimentary environments [17], [18], [19]. Two traits are noteworthy about rope builders among microorganisms involved in biostabilization; on the one hand they are typically pioneers, and on the other, they are comparatively very efficient in stabilizing their own habitat, in both terrestrial (*M. vaginatus* [20], [21]), and marine (*M. chthonoplastes* [22]) environments. It is thus reasonable to hypothesize on the basis of these correlations that rope building may somehow impart or promote their biostabilizing abilities. The key, however, is to find a mechanistic explanation as to why a rope should be superior to a mesh or a web of single filaments.

The mechanisms of sediment erosion are varied and complex as soon as sedimentary particles have been set in motion, but the conditions necessary for initial particle movement are relatively easy to model [23]. When a fluid such as water or air flows over a sedimentary bed, it transmits to it part of its momentum, exerting a tangential *shear stress*, τ_0_, over the contact surface that can cause movement of protruding particles and, aided by lift, the entrainment of sedimentary particles into the flow stream. Opposing this stress, we have the bed's shear strength, composed of gravity and internal cohesive forces. An erosion threshold occurs when stress overwhelms strength, a critical point commonly used to characterize the erodibility of sedimentary beds [24], [25]. Erosive and cohesive forces act on sedimentary particles and thus the “unit” of erosion is in principle a particle or a cohesive group of particles that the forces at play cannot break apart. The agency of microbes in biostabilization of sedimentary environments can result from their weaving of sedimentary particles and biological materials [8], from gluing those particles together with extracellular polymeric substances [25], or from particle cementation with biominerals formed *in situ* as a result of microbial metabolic activity [8]. In all these cases the net effect is an increase in the effective particle size subject to erosive forces. Alternatively, decreasing the surface roughness by means of copious amounts of polymeric substances can also ameliorate shear stress on the beds [18].

### A physical mechanism for fitness

**Figure 3:**
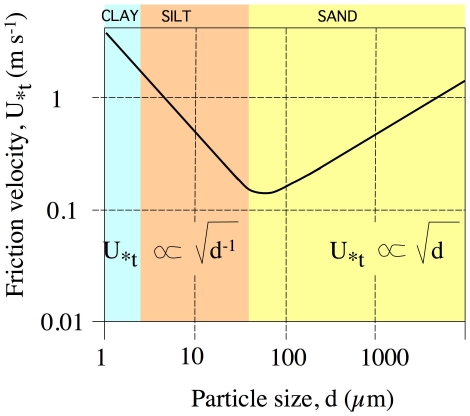
Erosivity by wind of sedimentary beds as a function of particle size. Threshold shear stress is estimated here as the *threshold friction velocity* (u_*__t_) above which particle movement will occur. *Friction velocity* (u_*_) is an integrated measure of the steepness of the velocity gradient and the roughness of the surface against flow, related to bed shear stress as τ_0_ = ρ_0_ (u_*_)^2^, where ρ_0_ is fluid density. Threshold friction velocities are a direct function of particle size for large particles where gravitational forces are dominant, but an inflexion point and minimum occurs around roughly 75 µm, where cohesive forces become more important and below which friction velocities actually decrease with particle size. The particle size dependence holds also for water erosion over sediments, and the values of the minimum range between 50 and 200 µm under a wide variety of conditions and sediment bulk densities [23].

By virtue of braiding fibers into yarns or ropes, three major overall parameters are increased: diameter, overall length span, and tensile strength. The tensile strength of a rope is actually less that the sum of the tensile strengths of all the fibers that compose it, since some strength is lost by fiber obliquity necessary for twisting [26]. In this sense, rope building does not represent an improvement in a microbial population's ability to stabilize sediment over the combined actions of single filaments, unless the sedimentary particles are so large that they cannot be spanned without a rope conformation. But this size (cm range) would imply gravel-sized sediments, which are practically non-erodible under most relevant (non-catastrophic) wind or current regimes anyway [24]. Thus, only the size increase remains a possible avenue for improvement. What is then the relationship between size of particles and the stability of sediments? Particle size is an important factor, since it affects particle weight and inter-particle cohesion (Fig. 3; adapted from reference [27]). For particles larger than 75–100 μm, gravity dominates the resistance to erosion so that the larger and heavier, the harder they are to lift. In this domain, microbial stabilization can be easily achieved through gluing or weaving particles together into a larger effective particle. But below 75–100 μm, in the silt and clay ranges, cohesive forces become increasingly important, sediments becoming more stable as particle size diminishes (Fig. 3). Gluing or weaving together of particles in this domain can actually make the substrate more prone to erosion, unless an opportunity is present to bind a number of particles large enough to exceed the critical size range. It is precisely this crucial size range, in the order of magnitude around 100 μm, that is reached during the formation of cyanobacterial ropes out of cyanobacterial filaments 4–6 μm thick. The differential effect of binding sedimentary particles with ropes becomes now clear: the “unit particle” of erosion reaches unavoidably the gravitationally dominated part of the curve regardless of the sedimentary particles size, as they are bound, entangled or glued to the common exopolysaccharide sheath of a 100 μm rope, with an immediate stabilizing effect upon contact with the biological agent.

The bimodal relationship between particle size and sediment stability thus imposes the necessity of “large body size” for stabilization by contact. We believe it is in this way that populations of rope-makers can expand laterally into virgin habitat in areas of high erosion potential, securing particle after particle along the path of an extending rope by sequentially affixing them to a macroscopic, practically non-erodible, organosedimentary complex particle. This can proceed independently of the length of periods or environmental stability, implying that rope-makers will obtain a selective advantage in high-energy, highly erodible habitats. Because of the pioneer condition, any competitive disadvantages involving self-shading or uptake inefficiency become moot, as they find no competition, a condition that might eventually be lost as the habitats becomes bio-stabilized and colonized by secondary populations. Such predictions are consistent with the successional dynamics observed in some ecosystems initially dominated by rope builders, such as stromatolites [8] or desert biological soil crusts [28].

## Materials and Methods

### Criteria for rope-formation in samples, microbial strains and public DNA sequences

Given the confusion between taxonomic systems for the cyanobacteria, the high level of entry errors found in public databases, and the facility with which rope building is lost upon cultivation, we included in our phylogenetic analyses only those sequences originating from cyanobacteria known to make ropes in a traceable and explicit manner. For field samples this meant sequences from isolated filaments obtained after direct micromanipulation of large ropes, either by us, or from the literature, when explicitly described as such in the original publication. For cultivated strains it meant isolates that form ropes presently, or those that have been documented to form ropes at one time, if an explicit reference was available. Table 1 lists the criterion for each sequence in detail.

**Table 1:** Sources for assignment of rope-building ability.

Sample/Strain	Genetic marker(s)	Correlated by
*M. vaginatus* field sample BW	*kai*C	direct observation
*M. vaginatus* field sample BW2	*kai*C	direct observation
*M. vaginatus* field sample BW3	*kai*C	direct observation
*M. vaginatus* field sample BW4	*kai*C	direct observation
*M. vaginatus* field sample BW5	*kai*C	direct observation
*M. vaginatus* field sample GR2	*kai*C	direct observation
*M. vaginatus* ASU LT04	*kai*C	direct observation
*M. vaginatus* PCC9802	*kai*C	description, ref. [5]
*M. vaginatus* OTA32c150 6A	16S rRNA	description, ref. [15]
*M. vaginatus* BS lac149 4B	16S rRNA	description, ref. [15]
*M. vaginatus* FI5MC4 c1821A	16S rRNA	description, ref. [15]
*M. vaginatus* OTA21c1521B	16S rRNA	description, ref. [15]
*M. chthonoplastes* SAG3898	*kai*C	direct observation
*M. chthonoplastes* Ney3 289	*kai*C	description, ref. [13]
*M. chthonoplastes* Ney3 407	*kai*C	description, ref. [13]
*M. chthonoplastes* Ney4 350	*kai*C	description, ref. [13]
*M. chthonoplastes* Ney5 361	*kai*C	description, ref. [13]
*M. chthonoplastes* K27FWS	*kai*C	description, ref. [13]
*M. chthonoplastes* FWS 17	*kai*C	description, ref. [13]
*M. chthonoplastes* NDN-1	*kai*C	description, ref. [4]
*M. chthonoplastes* PCC7420	*kai*C, 16S rRNA	description, ref. [4]
*M. chthonoplastes* GN5	16S rRNA	description, ref. [4]
*M. chthonoplastes* MEL1	16S rRNA	description, ref. [4]
*M. sociatus* SAG2692	*kai*C	description, ref. [4]
*M. sociatus* MPI96 MS KID	16S rRNA	description, ref. [4]
*M. steenstrupii* 73 2E	16S rRNA	description, ref. [15]
*M. steenstrupii* 94 2B	16S rRNA	description, ref. [15]
*M. steenstrupii* 52 2A	16S rRNA	description, ref. [15]
*M. steenstrupii* 150 3A	16S rRNA	description, ref. [15]
*M. steenstrupii* 177 7B	16S rRNA	description, ref. [15]

#### Sequencing and phylogenetic reconstruction

New sequences of 16S rRNA gene and *kai*C were obtained from either cultures or from field-collected samples (see Supplementary Table S1). DNA was extracted from culture samples using a kit (MoBio plant extraction kit). For field samples, ropes of filaments were excised from the habitat by micromanipulation under a dissecting microscope, and cleaned by dragging over agar surfaces. After morphological identification under the compound microscope, each rope was submerged in TE buffer (pH 8.0), frozen and thawed three times. Aliquots of each sample were used as a template for standard PCR amplification using previously described specific primers for the *kai*C gene [10] and 16S rDNA [4]. All PCR products were purified and sequenced on both strands using the same primers. New sequences were edited using Sequencher 4.1 (GeneCodes, Ann Arbor, MI) and then aligned with relevant sequences available from Genbank using ClustalX [29]. Phylogenetic analyses were performed as described previously [30]. Best fitting models for the two molecular sequence data sets were selected using the Akaike Information Criterion implemented in the program ModelTest v. 3.07 [31]: a GTR+I+Γ model for the 16S rDNA sequences; and a TVM+I+Γ model for *kai*C. Phylogenetic relationships were estimated with Bayesian inference using MrBayes v. 3.1 [32], in which two independent Markov chain Monte Carlo runs of 2–2.5×10^6^ generations were conducted using variable rate priors, with sampling every 1000 generations. Post burn-in sampled topologies (200) from each analysis were summarized as a majority rule consensus tree to obtain the posterior probability (PP) credibility interval for each clade.

## Supporting Information

Table S1 List of taxa included in phylogenetic analyses and Genbank accession number for molecular sequences.
(0.16 MB DOC)
