# Supplementary material for: The Evolution of a Capacity to Build Supra-Cellular Ropes Enabled Filamentous Cyanobacteria to Colonize Highly Erodible Substrates
Source: PLoS One. 2009 Nov 17;4(11):e7801. doi: 10.1371/journal.pone.0007801 (PMC2773439; doi:10.1371/journal.pone.0007801)
Supplement: Table S1 — List of taxa included in phylogenetic analyses and Genbank accession number for molecular sequences. (0.16 MB DOC) [file pone.0007801.s001.doc]

| **Taxon** | **Genbank accession,**  ***kai*C** | **Genbank accession,**  **16S rDNA** |
| --- | --- | --- |
|  |  |  |
| *Acaryochloris marina* MBIC11017 | AB120712 |  |
| *Anabaena variabilis* ATCC 29413 | NC_007413 |  |
| *Aphanizomenon flos-aquae* PCC 7905 |  | AY038035 |
| *Arthronema gygaxiana* UTCC 393 |  | AF218370 |
| *Arthrospira sp.* PCC 8005 |  | X70769 |
| *Arthrospira platensis* PCC 9108 |  | DQ393284 |
| *Arthrospira platensis* PCC 9223 |  | DQ393285 |
| *Calothrix desertica* PCC 7102 |  | AB075980 |
| *Calothrix parietina* DCC D550 | AF239754 |  |
| C*hlorogloeopsis fritschii* PCC 6718 | AF222603 |  |
| *Chlorogloeopsis fritschii* PCC 6912 |  | AB075981 |
| *Chroococcidiopsis sp.* PCC 6712 | AF222599 |  |
| *Chroococcidiopsis thermalis* PCC 7203 | AF239752 | AB039005 |
| *Cyanospira rippkae* PCC 9501 |  | AY038036 |
| *Cyanothece sp.* PCC 7418 |  | AF296872 |
| *Cyanothece sp*. PCC 7424 |  | AJ000715 |
| *Cyanothece sp.* PCC 8801 | AF442204 |  |
| *Cylindrospermum stagnale* PCC 7417 | AF222605 |  |
| *Dermocarpella* incrassata SAG 2984 (PCC 7326) |  | AJ344559 |
| *Fischerella muscicola* PCC 7414 |  | AB075986 |
| *Fischerella thermalis* PCC 7521 |  | AB075987 |
|  |  |  |
| *Geitlerinema sp*. PCC 7105 | AF222604 | X58360, AB039010 |
| *Geitlerinema sp.* PCC 9222 |  | AF317510 |
| *Gloeobacter violaceus* PCC 7421 | AF239755 | AY768399 |
| *Gloeocapsa* sp. PCC 73106 |  | AB039000 |
| *Gloeothece membranacea* PCC 6501 |  | X78680 |
| *Halomicronema excentricum* TFEP1 |  | AF320093 |
| *Halospirulina sp.* B1099 |  | AY426541 |
| *Halospirulina tapeticola* CCC Baja-95 Cl.2 |  | Y18791 |
| *Leptolyngbya* sp. PCC 6703 |  | AY768398 |
| *Leptolyngbya* sp. PCC 7104 |  | AY768404 |
| *Leptolyngbya* sp. PCC 7375 |  | AB039011 |
| *Leptolyngbya* sp. PCC 9207 |  | AF317506 |
| *Leptolyngbya* sp. PCC 9221 |  | AF317507 |
| *Leptolyngbya* sp. PCC 73110 |  | X84810 |
| *Lyngbya* sp. PCC 8106 | NZ_AAVU01000013 |  |
| *Lyngbya aestuarii* PCC 7419 |  | AY768409, AB075989 |
| *Microcoleus chthonoplastes* FWS-10 | AJ809755 | AJ809674 |
| *Microcoleus chthonoplastes* FWS-17 | AJ809741 | AJ809660 |
| *Microcoleus chthonoplastes* GN5-1 |  | AJ272597 |
| *Microcoleus chthonoplastes* K27-FWS | AJ809768 | AJ809687 |
| *Microcoleus chthonoplastes* MEL |  | AJ272596 |
| *Microcoleus chthonoplastes* NDN-1 | FJ646591 |  |
| *Microcoleus chthonoplastes* Ney3-289 | AJ809712 | AJ809631 |
| *Microcoleus chthonoplastes* Ney3-407 | AJ809744 | AJ809663 |
| *Microcoleus chthonoplastes* Ney4-350 | AJ809750 | AJ809669 |
| *Microcoleus chthonoplastes* Ney5-361 | AJ809751 | AJ809670 |
| *Microcoleus chthonoplastes* PCC 7420 |  | X70770 |
| *Microcoleus chthonoplastes* SAG3898 | FJ646590 | EF654089 |
| *Microcoleus sociatus* MPI 96MS KID |  | AF284809 |
| *Microcoleus sociatus* SAG2692 | FJ646592 | AF284808 |
| *Microcoleus steenstrupii* clone 173-2E (DG6-MC1) |  | AF355394 |
| *Microcoleus steenstrupii* clone 194-2B (FI6-MC1) |  | AF355385 |
| *Microcoleus steenstrupii* clone 152-2A (CSV2-N-1) |  | AF355383 |
| *Microcoleus steenstrupii* clone 150-3A (FB2-2) |  | AF355397 |
| *Microcoleus steenstrupii* clone 177-7B (JO1-MC1) |  | AF355396 |
| *Microcoleus vaginatus* BSA-1a (clone 149-4B) |  | AF355341 |
| *Microcoleus vaginatus* BW1 | FJ646582 |  |
| *Microcoleus vaginatus* BW2 | FJ646583 |  |
| *Microcoleus vaginatus* BW3 | FJ646584 |  |
| *Microcoleus vaginatus* BW4 | FJ646585 |  |
| *Microcoleus vaginatus* BW5 | FJ646586 |  |
| *Microcoleus vaginatus* GR2 | FJ646587 |  |
| *Microcoleus vaginatus* FI5-MC4 (clone 182-1A) |  | AF355350 |
| *Microcoleus vaginatus* LT04 ASU | FJ646588 | **GQ118962** |
| *Microcoleus vaginatus* MPI 98MV.JHS | GQ118963 |  |
| *Microcoleus vaginatus* OTA2-1 (clone 152-1B) |  | AF355349 |
| *Microcoleus vaginatus* OTA3-2 (clone 150-6A) |  | AF355342 |
| *Microcoleus vaginatus* PCC 9802 | FJ646589 | AF284803 |
| *Microcystis aeruginosa* PCC 7820 | DQ156152 |  |
| *Myxosarcina* sp. PCC 7312 |  | AJ344561 |
| Nodularia sphaerocarpa PCC 7804 |  | DQ185243 |
| Nodularia cf. spumigena IBASU-DS-13-00 | AY051293 |  |
| *Nodularia spumigena* PCC 73104 | AF222602 |  |
| *Nostoc* sp. PCC 9709, ‘AL#21 cyanosymbiont’ | AY373438 |  |
| *Nostoc* sp. PCC 7120 | AB071284 |  |
| *Nostoc* sp. PCC 7423 |  | DQ185242 |
| *Nostoc* sp. PCC 9709 | AY373437 |  |
| *Nostoc* cycadae | AB120713 |  |
| *Nostoc* linckia | AY051294 |  |
| *Nostoc linckia* IBASU-EC-I-2-20-98 | AY051313 |  |
| *Nostoc muscorum* CCAP1453-12 | AF239753 |  |
| *Nostoc punctiforme* ATCC29133 | CP001037 |  |
| *Oscillatoria sp.* PCC 6506 |  | AY768397 |
| *Oscillatoria sp.* PCC 7112 |  | AB074509 |
| *Oscillatoria sp.* ‘U-Stink’ |  | AY426542 |
| *Oscillatoria acuminata* PCC 6304 |  | AB039014 |
| *Oscillatoria corallinae* CJ1 SAG8.92 |  | X84812 |
| *Oscillatoria limnetica* 'Solar Lake' |  | U96443 |
| *Oscillatoria princeps* NIVA-CYA 150 |  | AB045961 |
| *Oscillatoria prolifera* UTEX B1270 |  | AB075993 |
| *Oscillatoria sancta* PCC 7515 |  | AF132933 |
| *Phormidium autumnale* UTEX 1580 |  | AY218830 |
| *Phormidium* sp. UTCC 487 |  | AF218376 |
| *Planktothrix* sp. PCC 7811 |  | AY768402 |
| *Plectonema boryanum* PCC 6306 |  | AY157926 |
| *Pleurocapsa* sp. PCC 7327 |  | Z82810 |
| *Pleurocapsa* sp. SAG 4-99 |  | AJ344564 |
| *Prochlorothrix hollandica* PCC 9006 |  | Z82782 |
| *Prochlorococcus marinus* MIT9313 | NC_005071 |  |
| *Prochlorococcus marinus* NATL1 |  | AF133834 |
| *Prochlorococcus marinus* PCC 9511 |  | AF180967 |
| *Pseudanabaena sp.* PCC 6802 |  | AB039016 |
| *Pseudanabaena sp.* PCC 6903 |  | AB039017 |
| *Pseudanabaena sp.* PCC 7367 |  | AB039018 |
| *Pseudanabaena sp.* PCC 7403 |  | AB075995, AB039019 |
| *Pseudanabaena tremula* UTCC 471 |  | AF218371 |
| *Scytonema hofmanni* PCC 7110 | AF222600 | AB075996 |
| *Spirulina sp*. CCC Snake P. Y-85 |  | Y18793 |
| *Spirulina sp.* MPI S4 |  | Y18792 |
| *Stanieria cyanosphaera* PCC 7437 |  | AF132931 |
| *Stanieria* sp. PCC 7301 |  | AB039009 |
| *Symploca atlantica* PCC 8002 |  | AB039021, AB075997 |
| *Synechococcus* sp. PCC 7002 | AB120711 |  |
| *Synechococcus* sp. PCC 7942 | AY120853 | AF132930 |
| *Synechococcus* sp. PCC 8806 |  | AF448077 |
| *Synechococcus* sp. PCC 8807 |  | AF448076 |
| *Synechococcus* sp. PCC 8916 |  | AF448071 |
| *Synechococcus* sp. WH8102 | NC_005070 |  |
| *Synechococcus* *elongatus* | AB071375 |  |
| *Synechococcus elongatus* PCC 6301 | AP008231 |  |
| *Synechocystis sp*. PCC 6308 |  | AB039001 |
| *Thermosynechococcus vulcanus* | AB121971 |  |
| *Tolypothrix sp*. PCC 7601 |  | AY768401 |
| *Trichodesmium erythraeum* IMS101 | NC_008312 | AB075999 |
| *Trichodesmium havanum* F34-5 |  | AF518770 |
| *Trichodesmium pelagicum* JWI1 |  | AF518769 |
| *Trichodesmium thiebautii* |  | AF091321 |
| *Xenococcus sp.* PCC 7305 | AF222601 |  |
|  |  |  |
